# Protective effect of royal jelly on the sperm parameters and testosterone level and lipid peroxidation in adult mice treated with oxymetholone

**Published:** 2014

**Authors:** Ensieh Zahmatkesh, Gholamreza Najafi, Vahid Nejati, Reza Heidari

**Affiliations:** 1 *Department of Histology and Embryology, Faculty of Science, Urmia University, Urmia, I. R. Iran *; 2 *Department of Anatomy and Embryology, Faculty of Veterinary Medicine, Urmia University, Urmia, I. R. Iran*; 3 *Department of Biochemistry, Faculty of Science, Urmia University, Urmia, I. R. Iran*

**Keywords:** *Mice*, *Oxymetholone*, *Royal jelly*, *Sperm*

## Abstract

**Objectives** **:** The aim of the present study was to evaluate protective effect of royal jelly on sperm parameters, testosterone level, and malondialdehyde (MDA) production in mice.

**Materials and Methods:** Thirty-two adult male NMRI mice weighing 30±2 g were used. All the animals were divided into 4 groups. Control group: received saline 0.1 ml/mouse/day orally for 30 days. Royal jelly group (RJ): received royal jelly at dose of 100 mg/kg daily for 30 days orally. Oxymetholone group: the received Oxymetholone (OX) at dose of 5 mg/kg daily for 30 days orally. Royal jelly+Oxymetholone group: received royal jelly at dose of 100 mg/kg/day orally concomitant with OX administration.

Sperm count, sperm motility, viability, maturity, and DNA integrity were analyzed. Furthermore, serum testosterone and MDA concentrations were determined.

**Results:** In Oxymetholone group, sperm count, motility as well as testosterone concentration reduced significantly (p<0.05), while significant (p<0.05) increases in immature sperm, sperm with DNA damaged, and MDA concentration were announced in Oxymetholone group in comparison with control group and Royal jelly+Oxymetholone group. RJ caused partially amelioration in all of the above- mentioned parameters in Royal Jelly+Oxymetholone group.

**Conclusion:** In conclusion, RJ may be used in combination with OX to improve OX-induced oxidative stress and male infertility.

## Introduction

OX (17b-hydroxy-2-hydroxymethylene-17a-methyl-5a-androstan-3-one) is a synthetic analogue of androgen and is a common treatment of aplastic anemia. Oxymetholone can stimulate hematopoiesis in bone marrow and under this condition, young and immature platelets are induced to a hypercoagulable state accompanied by an increase in platelet stickiness or adhesiveness (Kendall, 1948 ▶). 

Anabolic-Androgenic Steroid (AAS) compounds are used by athletes, prepubescent, and adolescents to improve athletic ability, appearance, or muscle mass. Therefore, administration of these compounds has increased significantly. Many of the athletes who use AAS believe that the side-effects are not serious or permanent. Many undesirable side-effects on the male reproductive function have been reported, but there is little information about how these compounds affect sexual behavior and reproductive system.

These side-effects are attributed to the negative endocrine feedback mechanisms induced on the hypothalamus, which follows the administration of the anabolic steroids, which then lower the overall fertility of bulls, rams, or bucks (Hafez et al., 2000 ▶). The main observed changes are oligospermia, azoospermia, and decreased testis size concomitant to a decrease in serum gonadotropin and testosterone levels (Orlande et al., 1964 ▶; Overly et al., 1984 ▶). Changes in these hormones can be reversed after discontinuing using anabolic steroids, but the long-term effects on the hypothalamus-pituitary-testicular axis are still unknown. However, certain residual abnormalities have been reported on the testicular morphology of healthy males six months after discontinuing the use of these steroids (Haupt and Rovere, 1984 ▶).

Anabolic agents particularly oxymetholone are used as ergogenic drugs to develop and strengthen muscles among athletes. Many researches on the psychological and physiological effects of this medicine have been conducted.

Royal jelly is gluten secreted by hypopharyngeal and submandibular glands of young worker bees of the genus Apismeliphera (Nakajima et al., 2009 ▶). It is an essential nutrient for young larvae bees and queens and has an important role in queen’s feeding.​ It has also been shown that royal jelly has different types of biological activity in various cells and tissues of animal models (Hashimoto et al., 2005 ▶). Royal jelly is mainly made of proteins, sugars, lipids (including sterols and fatty acids), and small amounts of mineral salts and vitamins (Hattori et al., 2007 ▶). 

Recent investigations indicated that these materials have different pharmacological activities such as antitumor and anti-microbial, and they can also dilate blood vessels, reduce blood pressure, stimulate growth, and increase resistance to infection. Royal jelly is an anti-hypercholesterolemic and shows anti-inflammatory activity which is why in more than 30 years it has been used commercially in pharmaceutical products, foods, and cosmetics (Kanbur, 2009 ▶). In the current study, the protective effects of royal jelly on sperm parameters, testosterone production, and lipid peroxidation in male mice treated with oxymetholone were evaluated.

## Materials and Methods

**Animals**

In this study, 32 adult male NMRI mice (30±2 g) were obtained from animal house of Faculty of Science, Urmia University, and were allowed to access water and food ad libitum under the controlled conditions of temperature (22±2 ^°^C), humidity (55±5%), and 12/12 hours light/dark.

**Drugs**

OX was used at a dose of 5 mg/kg. It was dissolved in saline before gavage. Royal jelly was used at a dose of 100 mg/kg.

**Drug treatment**

In this study, 32 adult male NMRI mice 8-9 weeks old were used. All animals were divided into 4 groups; each group consisting of 8 mice.

1. Control group (C): received saline 0.1 ml/mice/day orally for 30 days.
2. Oxymetholone group (OX): received oxymetholone 5 mg/kg/day orally for 30 days.
3. Royal Jelly group (RJ): animals were orally administrated 100 mg/kg royal jelly, daily for 30 days.
4. Royal Jelly+Oxymetholone group (RJ+OX): the mice received 5 mg/kg oxymetholone and 100 mg/kg royal jelly daily for 30 days.

**Sperm collection**

All animals were sacrificed with dislocation of the cervical vertebrae. Caudal epididymids was used to evaluate sperm parameters which were transferred into a 1 ml Human Tubal Fluid (HTF) medium with 4 mg/ml bovine serum albumin. The epididymids was cut into small pieces using surgical blades and incubated at 37 °C and 5% CO_2_ for 30 min.

**Sperm count**

The epididymal sperm count was determined by hemocytometry (Neubauer chamber) using the method described in the WHO manual (World Health Organization (WHO, 2010). Ten microliters of sperm suspension was transferred onto a slide Neubauer and sperms were counted.

**Sperm viability**

20 µl of spermatozoa from caudae pididymids were stirred (30 second at 37 °C) in 60 µl of Eosin&Nigrosin (E&N) stain, 5%-nigrosin and 4%-eosin-Y at ratio 3:1. Thereafter, smears were prepared and dried at room temperature. Sperms with stained cytoplasm in head were considered as dead sperms (Björndahl L et al., 2003 ▶).

**Sperm motility**

In order to observe mobility, 10 microliters of semen was placed on a glass slide and covered with a lamella. Using a light microscope with a magnification of ×40, the number of sperm with rapid progressive forward movement (RPFM), slowly progressive forward movement (SPFM), residual motion (RM), and motionless (ML) were counted in several microscopic fields of vision and percentage of motile and immobile sperms was obtained.

**DNA integrity assay,** **acridine orange (AO)**

AO staining was used to differentiate between denatured, single-stranded and native, double-stranded DNA regions in sperm chromatin (Morris et al., 2002 ▶).

Air-dried smears were fixed 12 hours in methanol-glacialacetic acid (3:1) at laboratory temperature. The slides were removed from the fixative and allowed to dry for a few min before staining with AO for 5 min at laboratory temperature. Staining solution was prepared daily from a stock solution consisting of 1 mg AO in 1000 mL of distilled water and stored in the dark at 4 °C. In order to prepare staining solution, 10 mL of the stock solution was added to 40 mL of 0.1 M citric acid and 2.5 mL of 0.3 M Na_2_HPO_4_·7H_2_O.

All solutions were maintained at laboratory temperature. After staining, the slides were gently rinsed in a stream of distilled water. Sperm cell heads with good DNA integrity had green fluorescence and those with diminished DNA integrity had orange-red staining (Juris et al., 2001 ▶).

**Evaluation of sperm maturity by aniline blue (AB)**

The sperm chromatin maturity refers to the replacement of sperm nuclear histones with protamine, which can be detected using some acidic stain such as acidic aniline blue (pH 3.5) (Fraser and Strzezek, 2007 ▶). AB staining was used to discern the histone-rich immature spermatozoa. Air-dried smears from samples of each animal were fixed in 3% buffered glutaraldehyde in 0.2 M phosphate buffer (pH 7.2) for 30 min at room temperature. Each smear was stained using 5% aqueous AB stain in 4% acetic acid (pH 3.5) for 5 min. Using light microscopy (Olympus Co, Tokyo, Japan), 200 spermatozoa were counted in each slide and unstained or pale-blue stained ones were considered as normal spermatozoa, whereas dark blue stained ones were considered as abnormal (Talebi et al ., 2008 ▶).

**Measurement of malondialdehyde (MDA) level**

MDA is the final product of fatty acid peroxidation, reacts with thiobarbituric acid (TBA) and produces a colored complex. The basis of spectrophotometric measurement of color is the reaction of TBA with MDA. In order to do this, 300 microliters trichloroacetic acid 10% was added to 150 microliters of sample and centrifuged at 1000 rpm for 10 min at 4 °C. Three hundred microliters of supernatant was transferred to a test tube with 300 microliters of thiobarbituric acid 67% and incubated at 100 °C for 25 min. Five min after cooling the solution, a pink color appeared because of MDA-TBA reaction and was evaluated using a spectrophotometer at a wavelength of 535 nm (Hosseinzadeh and sadeghnia, 2005 ▶). MDA was expressed as micromolar of MDA/gr tissue.

**Testosterone** **measurement**

After blood sampling, the serum was separated using a centrifuge (3000 g for 6 min) and kept at -70 °C until analysis of testosterone hormone. Serum testosterone concentrations were measured using immunoradiometric technique using WHO/Sigma Asso-RTGC-768/98 kits. 

**Statistical analysis**

All data in mentioned groups were analyzed using the one-way ANOVA by Tukey-Kramer test. The level of significance was considered as p<0.05.

## Results

**Sperm parameters**

**Figure1:**
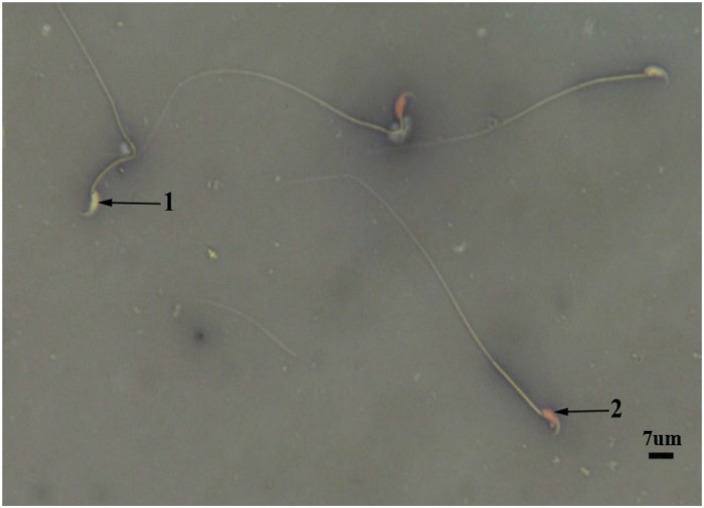
OX group**,** live sperm (1) with uncoloured head and dead sperm with pink or red (2) head (E&N, ×400).

**Figure 2:**
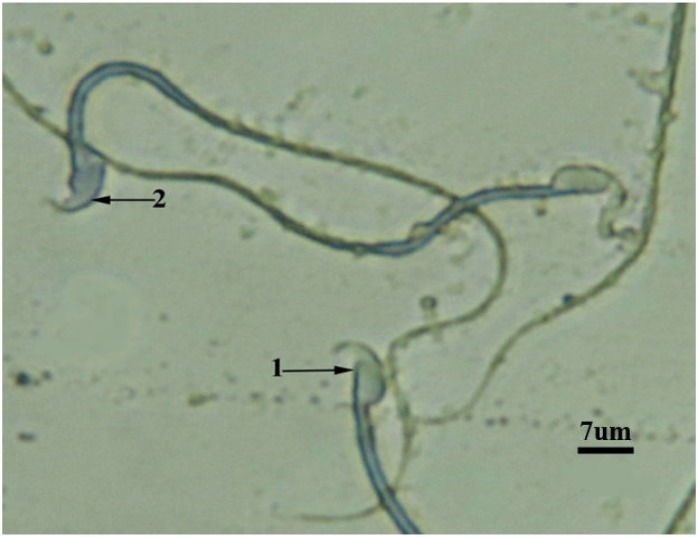
RJ+OX group: Sperm head with mature nuclei is light blue (1) and sperm head containing immature nuclear chromatin is dark blue (2) (AB, ×1000).

**Figure 3:**
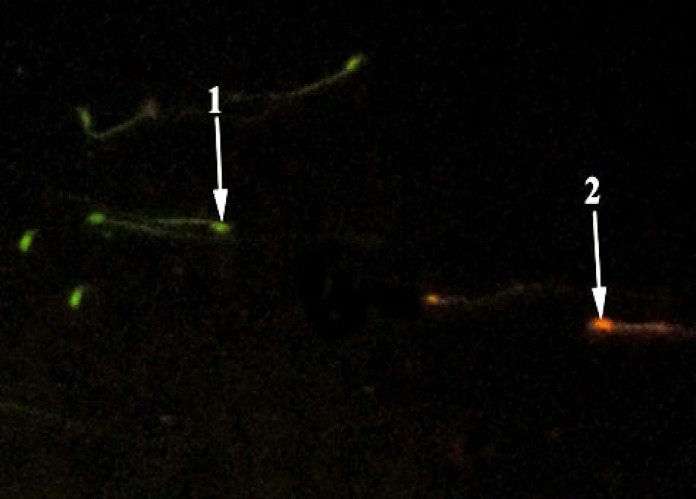
RJ+OX group: Sperm with normal DNA integrity had green fluorescence and those with diminished DNA integrity had orange-red staining (AO, ×400).

The results revealed that sperm count decreased significantly (p<0.05) in oxymetholone group. Royaljelly + Oxymetholone group revealed partially amelioration and enhancement in sperm count which is presented in Table1. There was no significant value in sperm viability among mentioned groups (Figure 1).In comparison with the control and royal jelly values, increased immature sperm and sperm with DNA damage was noticed in Oxymetholone-treated group (Figure 2 and 3). Royal Jelly+Oxymetholone group demonstrated a significant reduction (p<0.05) in immature sperms and sperm DNA damage (Table 2).

**Table1:** Protective effect of royal jelly on sperm parameters in mice treated with oxymetholone (mean±SEM)

**Group**	**Sperm count (× 10** ^6^ **)**	**Sperm viability** **(%)**	**Sperm motility (RPFM) (%)**	**Sperm motility (SPFM) (%)**	**Sperm motility** **(RM) (%)**	** Sperm motility (ML) (%)**
**Control**	38.80±1.42	89.06±1.24	66.83±1.90	16.53±0.76	10.23±0/57	7.06±.52
**Royal Jelly**	42.83±2.36	87.46±1.53	66.76±2.00	16.83±0.84	10.80±0.70	8.90±0.85
**Oxymetholone**	23.81±2.29^a^	82.46±1.56	54.16±1.33^b^	24.20±1.73^a^	10.86±0.67	11.13±1.21^b^
**Royal Jelly+Oxymetholone**	35.13±1.75	84.50±1.58	59.46±1.18	14.66±0.80	13.50±0.92	11.20±0.52^b^

a indicate statistically significant difference compared to the control, Royal jelly and Royal Jelly+Oxymetholone groups in each column (p<0.05);

b indicate statistically significant difference compared to the control and Royal jelly groups in each column (p<0.05).

**Table 2:** The effect of oxymethlone and royal jelly on DNA damage and sperm maturity in mice

**Group**	** AO** ^+^ ** (%)**	**AB** ^+^ ** (%)**
**Control**	15.20±1.17	9.46±1.15
**Royal JJelly**	14.40±0.55	6.76±0.97
**Oxymetholone**	24.13±1.12 a	21.73±1.51^a^
**Royal Jelly+Oxymetholone**	19.43±0.61	14.76±0.81

AO^+^: Acridine-orange positive (sperm with DNA damage), AB^+^: Aniline blue positive (immature sperm);

a indicate statistically significant difference compared to the control, Royal jelly and Royal Jelly+Oxymetholone groups in each column (p<0.05).

Regarding sperm motility, RPFM was decreased significantly (p<0.05) in OX group in comparison with the C and RJ group. Furthermore, SPFM and RM as well as ML increased in OX group. Daily administration of royal jelly caused a significant (p<0.05) increase in the sperm motility type RPFM in comparison with OX group. 

**Evaluation of MDA**

OX-induced lipid peroxidation in the testis as revealed by significant (p<0.05) elevation of MDA in OX group compared with C group and RG. MDA content in RG-OXG were lower than those in the OX group. Therefore, RJ administration caused partially decline of MDA in RJ+OX group. 

**Testosterone assessment**

Oxymetholone administration in OX group compared with C and RJ group caused reduction of testosterone level. Accordingly, cases in RJ+OX group revealed remarkable increase (p<0.05) of testosterone level in blood circulation in comparison with OX group (Table 3).

**Table 3:** The effect of oxymetholone and royal jelly on testis lipid peroxidation and testosterone production in mice

**Group**	**MDA (µmol/gr tissue)**	**Testosterone (ng/ml)**
**Control**	392.69±16.93	3.97±0.16
**Royal Jelly**	384.80±22.30	4.25±0.24
**Oxymetholone**	623.72±22.64^a^	2.83±0.21^a^
**Royal Jelly+Oxymetholone**	477.30±11.02^c^	3.73±0.88

a indicate statistically significant difference compared to the control, Royal jelly and Royal Jelly+Oxymetholone groups in each column (p<0.05).

c indicate statistically significant difference compared to the control, Royal jelly and Oxymetholone groups in each column (p<0.05).

## Discussion

In fact, synthetic anabolic-androgenic steroids such as oxymetholone act similar to androgen hormones. In rats, anabolic steroids have marked effects on the testes and accessory sex organs, causing arrest of spermatogenesis as well (Kincl et al., 1965 ▶). Sperm count is one of the most sensitive tests for spermatogenesis (Wyrobek and Bruce, 1975 ▶) and fertility evaluation (Wyrobek and Bruce, 1975 ▶; Whorton et al., 1977 ▶; Sandifer et al., 1979 ▶; SanchezPena et al., 2004 ▶). In this study, in the group treated with oxymetholone, significant decrease in sperm count was observed. This decrease in sperm count may be due to these anabolic effects. 

Sperm viability plays an important role in determining male reproductive success in sexually promiscuous species (Dziuk, 1996 ▶; Birkhead et al., 1999 ▶; Hunter and Birkhead, 2002 ▶). Both activated and hyperactivated motilities are necessary for fertility in several mammalian species and some of the signaling molecules regulating these processes have been identified (Quill et al., 2003 ▶; Esposito et al., 2004 ▶; Nolan et al., 2004 ▶; Chang and Suarez, 2011 ▶; Lishko et al., 2011 ▶; Zeng et al., 2011 ▶). In the current study, oxymetholone reduced sperm motility.

Growth factors play an important role in regulating the paracrine and autocrine function of testis in highly proliferative cells. In the testis, IGF-I is secreted from Sertoli and Leydig cells under the control of FSH and LH, respectively (Lejeune et al., 1996 ▶). IGF-I receptor has been identified in spermatogonia, spermatocytes, spermatids, and spermatozoa (Henricks et al., 1998 ▶; Vannelli et al., 1988 ▶). IGF-I has been suggested to be an important factor in germ cell development, maturation, and motility of the spermatozoa (Glander et al., 1996 ▶; Henricks et al., 1998 ▶; Vickers et al., 1999 ▶). 

Since anabolic steroids such as OX affect the hypothalamus-pituitary-testicular axis and reduce gonadotropin hormones, it can be concluded that, OX decreased sperm maturity and motility. However, in the current study, oxymetholone caused the increased percentage of sperm maturation.

Moreover, oxymetholone decreased the amount of plasma testosterone and the decline may be due to that anabolic steroids like oxymetholone which occupies androgenic receptors and causes negative feedback responses to the brain and hypothalamic-pituitary-gonadal axis to inactivate, resulting in the reduction of testosterone levels. The sperm chromatin structure assay is an independent predictor to indicate successful pregnancy (Bungum et al., 2011 ▶). During spermatogenesis, sperm DNA becomes condensed and the majority of histones are replaced with protamines (Carrell et al., 2007 ▶). DNA damage in sperm can result in impaired fertility or adverse effects on early embryonic development (Singerand Yauk, 2010). DNA damage may be fixed as mutations in sperm DNA and be inherited and results in genetic disorders. In the current study, oxymetholone increased DNA damage percentage.

In this study, we used a dose of 100 mg/kg royal jelly and its effect on the production of testosterone and sperm parameters was investigated. Royal jelly caused an increase in sperm count, maturation, motility, and plasma testosterone levels. Moreover, it caused decreased DNA damage percentage which could be due to the fact that royal jelly mainly includes proteins, sugars, lipids, vitamins, and free amino acids (Takenaka, 1982 ▶). It has been shown that it possesses several pharmacologic activities including vasodilative and hypotensive and increases growth rate. It also has disinfectant action, antitumor activity, anti-inflammatory, antioxidative, and scavenging ability, hypoglycemic and wound healing activity, immunomodulatory, and estrogenic activity (Shimoda et al., 1978 ▶; Fujii A et al., 1990 ▶; Sver L et al., 1996 ▶). Moreover, it was demonstrated that the protein fractions in RJ have high antioxidative activity and scavenging ability against free radicals such as superoxide anion radical, DPPH (1,1-diphenyl-2-picrylhydrazyl) radical, and hydroxyl radical (Nagai and Inoue, 2004 ▶). Natural compounds such as RJ with antioxidant and immunomodulatory activity might be useful in the prevention of sperm toxicity side-effects induced by Cisplatin.

The amino acid content of both honey and royal jelly may play a role as well by enhancing acrosome reaction, sperm motility, or improving fertilization (Renardet al .,1996 ▶) and enhancing sperm motility by the short-chain fatty acids specific of royal jelly, especially 10-hydroxy-2-decenoic acid (Comhaireet al., 2000 ▶). Sibel silica et al. studied the effect of royal jelly with the dose of 50-100 mg/kg on rat sperm parameters that showed the similar results (Ahmed et al., 2008 ▶). However, Anshu yang and colleagues examined the royal jelly effects with 200, 400, and 800 mg/kg doses on the reproductive performance of male rats for 4 weeks in 2011 which had effects such as increased weight, but number of sperm did not have any significant change and also an increase in the levels of estrogen, LH, FSH, and decrease in testosterone was observed (Anshu Yang et al., 2012).

Recently, greater emphasis has been placed on understanding the role of oxidative stress in the development of various testicular dysfunctions in experimental animals and male infertility in humans (Aitken, 1994 ▶; Aitken, 1995 ▶). During the course of lipid peroxidation, several products are produced which among them MDA and isoprostane F2α are the most important ones (Sanockaand and Kurpisz, 2004 ▶; Meagher and FitzGerald, 2000). The most common biomarker used to investigate lipid peroxidation in the sperm is MDA which is measured by thiobarbituric acid test (TBA) (Gomez et al., 1998 ▶).

The study of Fraczek et al. showed that malondialdehyde levels of seminal plasma in pathologic conditions such as asthenozoospermia is higher than normospermic mode (Fraser and Strzezek, 2007 ▶). In this study, oxymetholone led to an increase in production of MDA and royal jelly reduced MDA due to its antioxidant property.

Oxymetholone has negative effect on male reproductive system. However, royal jelly has partially positive effects on sperm parameters and peroxidation of fatty acids and accordingly has a positive role in fertility male mice.
